# Correlation Analysis Among the Chemical Composition and Cytotoxic and Antioxidative Activities of a *Tessaria absinthioides* Decoction for Endorsing Its Potential Application in Oncology

**DOI:** 10.3390/plants13213062

**Published:** 2024-10-31

**Authors:** Lourdes Inés Pascual, Lorena Luna, Roxana Elizabeth González, Javier Esteban Ortiz, Luciano Gomez-Gomez, Osvaldo Juan Donadel, María Belén Hapon, Gabriela Egly Feresin, Carlos Gamarra-Luques

**Affiliations:** 1 Instituto de Medicina y Biología Experimental de Cuyo (IMBECU), Universidad Nacional de Cuyo, CCT Mendoza CONICET, Ruiz Leal s/n, Mendoza 5500, Provincia de Mendoza, Argentina; lourdes.p.97@gmail.com (L.I.P.); bhapon@mendoza-conicet.gob.ar (M.B.H.); 2 Facultad de Ciencias Exactas y Naturales, Universidad Nacional de Cuyo, Padre Jorge Contreras 1300, Parque General San Martín, Mendoza 5502, Provincia de Mendoza, Argentina; gonzalez.roxana@inta.gob.ar (R.E.G.); luchog120@gmail.com (L.G.-G.); 3 Facultad de Ingeniería, Instituto de Biotecnología, Universidad Nacional de San Juan, CCT CONICET San Juan, Av. Libertador General San Martin 1109 Oeste, San Juan 5400, Provincia de San Juan, Argentina; lorenaluna@unsj-cuim.edu.ar (L.L.); jortiz@unsj.edu.ar (J.E.O.); gferesin@unsj.edu.ar (G.E.F.); 4 Instituto Nacional de Tecnología Agropecuaria (INTA), Estación Experimental La Consulta, Ex Ruta 40 km 96, La Consulta, San Carlos 5567, Provincia de Mendoza, Argentina; 5 Facultad de Química, Bioquímica y Farmacia, Instituto de Investigaciones en Tecnología Química (INTEQUI-CONICET), Universidad Nacional de San Luis, Área de Química Orgánica, Almirante Brown 1445, San Luis 5700, Provincia de San Luis, Argentina; odonadel@gmail.com; 6 Facultad de Ciencias Médicas, Universidad Nacional de Cuyo, Centro Universitario, Mendoza 5502, Provincia de Mendoza, Argentina

**Keywords:** phytomedicine, polyphenols, tessaric acid, cancer, chemical marker

## Abstract

Historically, botanical preparations have been used to improve human health. Their active ingredients are influenced by multiple factors such as intraspecies variations, environmental conditions, collection time and methods, and the part of the plant used. To ensure the efficiency and safety of these herbal drugs, qualitative and quantitative analyses are required. A *Tessaria absinthioides* decoction (DETa) was reported as having hypocholesterolemic, anti-inflammatory, cytotoxic, antitumor, and antioxidative properties. This work aimed to analyze DETa by correlating its chemical composition with cytotoxic and antioxidative properties, with the aim of promoting research on it as an anticancer agent. DETa collections (2017, 2018, 2019, and 2022) were analyzed by UHPLC-DAD, UHPLC-DAD-FLD, and UPLC-MS/MS; cytotoxicity was assessed on the MCF-7 breast cancer cell line; antioxidative capacity was evaluated by the DPPH and FRAP methods; and correlation analysis was used to determine biological and chemical markers. The results provide evidence that biological activities were consistent across the collections. Among the quantified compounds, apigenin, naringin, gallocatechin gallate, ginnalin A, myricetin, epicatechin, OH-tyrosol, quercetin, and chlorogenic, tessaric, *p*-coumaric, vanillic, caffeic, caftaric, ellagic, and rosmarinic acids correlated as bioactive and chemical markers. Moreover, tessaric acid could be established as a species marker. Altogether, these findings add relevant information to DETa properties, encouraging further exploration of its potential application as an anticancer botanical.

## 1. Introduction

Historically, plant-based preparations have been used to enhance human health. Plants offer a multitude of ways to harness their therapeutic properties. The most common application is in homemade remedies, such as herbal teas. Additionally, the vast chemical variety in plants presents endless possibilities for new drug development. Systematically searching for and documenting traditional medicine knowledge is crucial to identify bioactive compounds that support human health, are backed by various scientific disciplines, and possess commercial significance. However, native plants used as “natural remedies” for various ailments may be toxic. To confirm the presence of bioactive molecules that align with traditional uses and to potentially reveal new properties, a range of standardized bioassays are conducted. Ensuring the efficacy and safety of these herbal drugs requires qualitative and quantitative analyses, which includes standardizing the active ingredients, authenticating botanical materials, and conducting scientific research in order to confirm the health benefits and to understand the action mechanisms of various botanicals [1]. Moreover, maintaining integrity and trust in botanical products is essential to ensure that they are safe and effective for consumer use [2].

The chemical profile of preparations obtained from the same plant may vary depending on the solvent(s), the temperature, and the extraction time applied. Additionally, the active ingredients are influenced by multiple factors such as intraspecies variation, environmental conditions, season of the year, time and methods of collection, geographical location, and the specific part of the plant used. Therefore, managing the natural variations in botanicals and employing standardized extraction procedures can help to produce extracts with a consistent composition [3].

Cancer ranks among the leading causes of death and contributes to a significant global health burden due to the substantial costs associated with managing the disease for those affected. Cancer represents a significant challenge for society, public health, and the economy in the 21st century. According to the global cancer statistics based on updated estimates from the International Agency for Research on Cancer (IARC), in 2022, there were an estimated 20 million new cases of cancer and 9.7 million deaths from cancer [4].

Regarding their medicinal properties, it is a well-recognized fact that some botanical preparations have anticancer effects. The bioactive phytochemicals present in these preparations can modulate signaling cascades responsible for cell proliferation, apoptosis, angiogenesis and cell migration–invasion processes. Modulation of these signaling pathways, which are implicated in tumoral growth and metastasis, would explain the anticancer effects of the extracts. In a recent review, Chandra et al. [5] revealed that plants and their phytochemicals could be crucial in fighting a range of cancers, including those of the oral cavity, breast, lungs, cervix, colon, stomach, and liver. Certain bioactive agents are known to stimulate biological responses that may contribute to controlling cancer cells. Among the natural compounds that can trigger anticancer activity, polyphenols have been reported to inhibit tumor growth, metastasis, and angiogenesis [6].

Botanical derivatives have been used as lead compounds for the development of new anticancer drugs worldwide. In consequence, it is expected that novel therapeutics derived from medicinal plants can be developed to treat patients with cancer. In addition to their effective activity against cancer cells, botanical preparations are recognized as inducing fewer side effects compared with current anticancer medicine due to their natural origin [7]. The beneficial effects in cancer described for botanicals can be attributed to the synergistic interaction among the phytochemicals present into the preparations [8].

Additionally, the anti-oxidative activity of botanical extracts is well known, and this is recognized as conferring health-promoting effects. It is widely accepted there is a strong correlation between the phenolic content of plant-derived extracts and their ability to prevent oxidative damage in biological systems. Different studies have shown that a plant’s complex matrices are more effective at preventing oxidative damage at the biological level than isolated phytochemicals [9,10]. There is substantial evidence linking oxidative stress to human diseases. In particular, reactive oxygen species (ROS) are reported to be involved in all stages of cancer, contributing to cancer progression and metastasis. Therefore, antioxidative activity can significantly influence the course of this disease, and the inhibition of ROS production is considered a crucial strategy for preventing the spread of cancer [11].

The study of botanical preparations for medicinal purposes requires determining the correlation between its chemical composition and the attributed biological properties, adding value to these botanicals as a source of bioactive molecules that can be applied in a wide range of manufactured products [12]. In particular, there are numerous studies planned for the characterization of biological preparations by the correlation between their phytochemical content with their cytotoxicity and/or antioxidative properties [13,14,15].

*Tessaria absinthioides* (Hook. & Arn.) DC, Asteraceae, popularly called “pájaro bobo”, is a native plant of Bolivia, Chile, Uruguay, Paraguay, and northern and central Argentina. It has been widely used by native populations from Argentina and Chile because of its medicinal properties as a hypocholesterolemic, hypoglycemic, and anti-inflammatory agent and in the treatment of digestive disorders [16,17,18]. In previous reports, an aqueous preparation of *T. absinthioides* exhibited selective cytotoxicity in cancer cells lines, affecting human glioblastoma, cervicouterine, mammary, and colorectal cancer cells. The oral administration of the aqueous extract induced antitumoral effects, improving the overall survival of mice with colorectal cancer and reducing the tumoral growth of murine melanoma [19,20]. Additionally, the antioxidative activity of the *T. absinthioides* aqueous preparation was demonstrated in vitro [21] and in vivo on the ApoE-KO mice [22].

The aim of the current work is to analyze the *T. absinthioides* decoction DETa using a systematic correlation analysis, considering its chemical profile, cytotoxic activity in MCF-7 cells, and antioxidative properties, to promote further research because on its anticancer potential.

## 2. Results

### 2.1. Chemical Analysis

Using UHPLC-DAD, UHPLC-DAD-FLD, and UPLC-MS/MS, a total of thirty-two compounds were quantified, of which 96.88% (*n* = 31) were phenolic acids, flavonoids, phenylethanoids, and stilbenes. Additionally, the eremophilane sesquiterpene compound known as tessaric acid was identified and quantified.

A percentage of 70.97 (*n* = 22) of the total quantified compounds was identified across the four collections analyzed. Their concentrations are presented as the mean ± standard deviation and categorized by year, as shown in Table 1; the DETa phytochemicals occasionally quantified in some collections are shown in Supplementary Table S1.

Out of the twenty-two compounds identified and quantified in the four analyzed DETa collections, only six had been previously reported. Gomez et al. [21] described ginnalin A and chlorogenic, vanillic, and tessaric acids in lyophilized aqueous preparations of *T. absinthioides*, while hesperetin and quercetin were described in methanolic and hydroethanolic extracts [17,23,24]. In this study, the analysis of DETa collection samples resulted in the identification and quantification of sixteen phenolic compounds in *T. absinthioides* for the first time, including eight flavonoids: apigenin, catechin, epicatechin, gallocatechin gallate, luteolin, myricetin, naringin and quercetin-3-glucoside; six phenolic acids: caffeic, caftaric, ellagic, ferulic, *p*-coumaric, and rosmarinic acid; the phenylethanoid OH-tyrosol; and the stilbene *trans-*piceatannol.

The most abundant compounds were tessaric acid (276.50 ± 224.31 µg/mL mean; range 559.50–29.80 µg/mL), rosmarinic acid (37.62 ± 38.58; 93.22–5.14 µg/mL), naringin (23.52 ±17.47; 44.43–1.92 µg/mL), caftaric acid (23.26 ± 35.02; 75.74–3.82 µg/mL), querce-tin-3-glucoside (22.33 ± 21.92; 45.32–1.47 µg/mL), and chlorogenic acid (10.71 ± 1.96; 13.10–8.37 µg/mL). Additionally, the following compounds were detected and quantified: luteolin, caffeic acid, ginnalin A, ellagic acid, catechin, apigenin, myricetin, and hesperetin (See Table 1). The most abundant compounds identified (tessaric acid, chlorogenic acid, ginnalin A, and hesperetin) are consistent with those reported in previous studies on *T. absinthioides* [17,21,23,24]. In addition, ten compounds were detected occasionally in some of the collections; these compounds were phenolic acids such as synaptic, syringic, and trans-cinnamic acid; the phenylethanoid tyrosol; and flavonoids such as kaempferol-3-glucoside, naringenin, phloridzin, procyanidin B1, procyanidin B2, and rutin (See Supplementary Table S1). These variations in the chemical profiles among the DETa collection samples may be attributed to the edaphoclimatic conditions of each year, such as temperature, radiation, rainfall, and soil characteristics, among other factors.

### 2.2. Determination of Total Phenolic Content and Antioxidative Activity

For the decoction samples of *T. absinthioides* harvested in different years, the total phenolic content (TPC) was determined using the Folin–Ciocalteu method, and the antioxidative activity was measured using the DPPH decolorization method and the FRAP assay. The quantification of polyphenols can be considered useful for estimating antioxidative activity [25] due to the role that these compounds play in this bioactivity. Numerous publications have applied the total phenolic content assay using the Folin–Ciocalteu reagent and an electron-transfer-based antioxidative capacity assay (e.g., FRAP, TEAC, etc.) and often find excellent linear correlations between the total phenolic profiles and the antioxidative activity. This is not surprising if one considers the similarity of the chemistry between the two assays [26].

**Figure 1:**
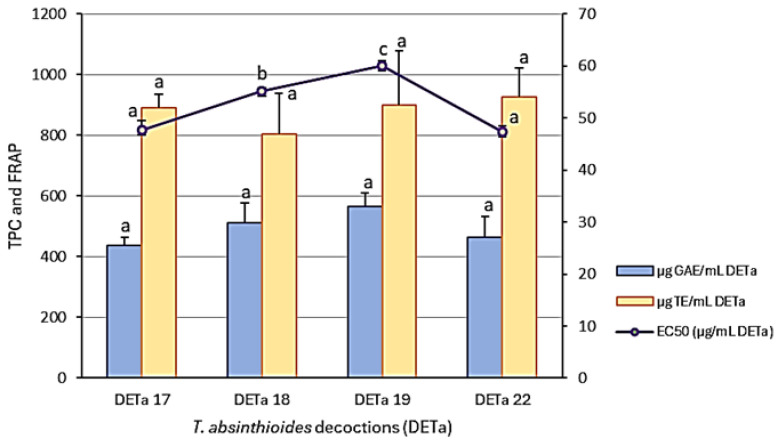
Total phenolic content (blue bars), FRAP (orange bars), and EC50 of the DPPH assay (purple line) of *T. absinthioides* decoction (DETa) samples. Results are expressed as the mean ± SD (standard deviation). Different letters indicate significant difference among samples, as determined by the Tukey test (*p* < 0.05).

These results are shown in Figure 1. Various studies have evaluated the antioxidative activity of this species [21,22,23,24]. In this case, the different DETa collection samples did not show significant differences in the TPC and FRAP assays. However, the results of the DPPH radical decolorization assay, expressed as the EC50 (µg/mL), showed lower values for the DETa 2017 and DETa 2022 samples, indicating a higher antioxidative capacity. Regarding the TPC content, there are no significant differences among the evaluated samples, with the highest value recorded in DETa 2019 (563.07 ± 44.15 µg GAE/mL) and the lowest in DETa 2017 (436.65 ± 25.57 µg GAE/mL). The TPC values for DETa 2018 and DETa 2022 were 511.31 ± 63.19 and 461.84 ± 70.62 µg GAE/mL, respectively (ANOVA, Tukey test; significance *p* < 0.05). Similarly, Rey et al. [23] reported a high total phenolic content in a 10% *w*/*v* decoction of *T. absinthioides* collected from the San Juan province in Argentina.

Concerning the antioxidative potential, no significant differences (ANOVA, Tukey test; *p* ≤ 0.05) were observed among the different years of collection. The reducing power, assessed by the FRAP assay, remained consistent across all the samples, suggesting a possible link to bioactive compounds that reduce iron, with values ranging from 802.07 to 925.85 µgTE/mL. Meanwhile, the DPPH assay demonstrated an effect in free radical scavenging, with DETa 2018 (55.13 ± 0.68 µg/mL) and DETa 2019 (60.07 ± 0.85 µg/mL) exhibiting significant differences when compared with DETa 2017 (47.71 ± 1.73 µg/mL) and DETa 2022 (47.27 ± 1.23 µg/mL). The minor variations observed may be attributed to factors affecting plant physiology, including radiation and edaphoclimatic conditions. The stable antioxidative capacity of the species *T. absinthioides* over various collection years is substantial for the development of potential phytopharmaceuticals. In this work, determinations were made directly from DETa. Although the TPC values obtained are lower than those obtained in other aqueous preparations, such as green tea decoctions (908.46 µg GAE/mL), a common beverage with widely accepted medicinal properties and recognized for its high levels of polyphenols [27,28], they still fall within the range of high content for this type of compounds.

The antioxidative properties of *T. absinthioides* aqueous preparations have been previously documented through in vitro and in vivo studies. From a comparison of lyophilized decoctions from San Juan (Argentine), Mendoza (Argentine), and Antofagasta (Chile), Gomez et al. [21] reported strong antioxidative capability from all the samples studied using the DPPH assay, with IC_50_ values of 42, 41.6 and 43 µg/mL. These values were similar to the quantifications obtained in DETa 2017 and 2022, indicating a similar antioxidative potency.

Moreover, Quesada et al. [22] demonstrated a decrease in lipid peroxidation and an enhancement in the total antioxidative status in mice plasma after the oral administration of an aqueous extract. Meanwhile, Rey et al. [23] reported that in a hypercholesterolemic model using adult male rats, the consumption of a *T. absinthioides* decoction led to a decrease in cholesterol levels, which was associated with the identified antioxidative compounds. Collectively, these findings affirm the significant antioxidative potential of *T. absinthioides* aqueous preparations and endorse their use in vivo.

### 2.3. Determination of DETa Cytotoxic Acivity

**Figure 2:**
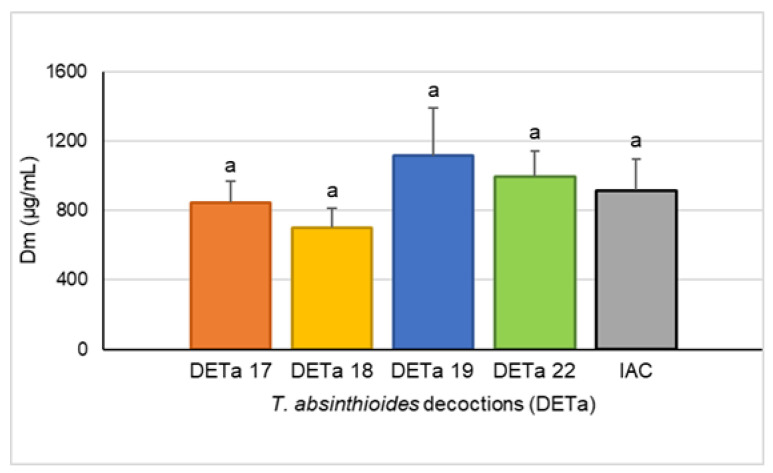
Cytotoxicity of DETa collections discriminated by year. Dm: median-effect dose. IAC: interannual cytotoxicity (calculated as the mean of DETa 2017, 2018, 2019, and 2022 cytotoxicity). Assays were performed in triplicate, and the means ± SD were compared by ANOVA, followed by the Tukey’s multiple comparisons test. a: indicates that no significant differences were found between the groups (*p* ≤ 0.05).

To describe and compare the cytotoxicity induced by the 2017, 2018, 2019, and 2022 DETa collections, the MTT assay was used on the MCF-7 human breast adenocarcinoma cell line. This is a colorimetric test that is commonly used to determine cellular metabolic activity, and it provides information about cell proliferation, viability, and toxicity under different paradigms of treatment. The cytotoxic potency of DETa was expressed by the median-effect dose (Dm), which is the concentration of treatment that is able to reduce cell proliferation by 50%. The calculated Dm mean for the four DETa collections samples was 915.64 ± 182.13 µg/mL (interannual cytotoxicity; IAC), and there were no significant differences between each DETa year (ANOVA, Tukey; *p* ≤ 0.05). Moreover, while the highest cytotoxicity (lowest dose that reduces 50% of the proliferation) was measured for DETa 2018 with a, Dm of 700.68 ± 111.36 µg/mL, the lowest was induced by DETa 2019 (Dm = 1116.00 ± 277.71 µg/mL). DETa 2017 and 2022 induced cytotoxicity with Dm values of 845.72 ± 123.45 and 997.5 ± 143.39, respectively (Figure 2).

Cytotoxicity of the aqueous preparation of *T. absinthioides* against the MCF-7 cell line has been previously reported [19]. This study employed an alternative preparation method to preserve the yield percentages and phytochemical concentrations for each sample to ensure the quality of DETa and to facilitate further correlation analyses.

In terms of comparative potency, DETa cytotoxicity result was higher than some of the oncologic treatments prescribed by traditional Chinese medicine (TCM), such as the Taohong Siwu Decoction (TSD), Shugan Liangxue Decoction (SLD), and a new TCM formula T33. These formulas, orally administered, are used clinically as adjuvant treatments on breast cancer due to their ability to increase the anticancer effects and control the side effects induced by chemotherapy [29]. When these treatments were tested against the MCF-7 breast adenocarcinoma Luminal A-like cell line, the reported Dm values were 5 mg/mL for T33 [30], 12 mg/mL for TSD [31], and 14.60 mg/mL for SLD [32]. The Dm values obtained in this study for the DETa collections ranged from 1.1–0.700 mg/mL, suggesting that they could be used as a botanical preparation for breast cancer treatment.

### 2.4. Relationships Between the Bioactive Compound Content and Biological Properties

To determine the biological and chemical marker correlations, cytotoxic and antioxidant activities of the twenty-two compounds identified in the four DETa collections were analyzed by Pearson analysis (Supplementary Table S2).

When the relations among particular phytochemicals were considered, the multivariate analysis showed evidence of sixteen very strong correlations with an r ≥ 0.95. Ellagic acid showed high correlations with epicatechin (r = 0.98), gallocatechin gallate (r = 1), myricetin (r = 0.99), naringin (r = 0.95), OH-tyrosol (r = 0.97), and rosmarinic acid (r = 0.98). Similarly, absolute correlations (r = 1) were found between caffeic–caftaric acids and myricetin–rosmarinic acid.

Regarding the compound–cytotoxicity correlation, thirteen compounds presented positive correlation values (see Supplementary Table S2). While six of them—caffeic, caftaric, ferulic acids, ginnalin A, naringin and quercetin-3-glucoside—evidenced very weak or weak correlations (Pearson r values ranging from 0.01 to 0.39), another three compounds showed moderate correlations (Pearson, r: 0.40–0.59)—hesperetin, *trans*-piceatannol, and vanillic acid. Finally, the concentration of four compounds strongly or very strongly correlated with cytotoxicity (Pearson, r: 0.60 to 1): apigenin, chlorogenic acid, *p*-coumaric acid, and tessaric acid. Compounds with Pearson´s correlation “r” values greater than 0.5 are summarized in Table 2. In this last group, special consideration should be given to chlorogenic acid (r = 0.98) and tessaric acid (r = 0.89), which evidenced the highest correlation values calculated (Pearson´s very strong correlation).

The majority of the analyzed compounds that demonstrated a positive correlation with cytotoxicity have been widely reported for their effects on cancer cells. Previous in vitro studies reported no cytotoxic effects of tessaric acid on A2780 (ovarian), A549 (lung), HeLa (uterine cervix), SW1573 (lung), T47D (breast), or WiDr (colon) cancer cell lines [33].

Among the five compounds that showed a Pearson´s correlation with cytotoxicity (r > 0.5), chlorogenic acid (Pearson, r = 0.98) stands out as an abundant dietary phenolic with demonstrated anticancer effects in various types of cancer cells by arresting cell proliferation, promoting apoptosis, and facilitating intracellular DNA impairment [34]. Apigenin (r = 0.74), a common flavonoid in the plant kingdom, has demonstrated anticancer properties in both in vitro and in vivo models [35]. Moreover, *p*-coumaric acid (r = 0.73), derived from green propolis, has been reported to target melanoma cancer cells [36], while vanillic acid (r = 0.54) has been shown to affect tumor growth on xenograft colorectal cancers [37].

When the antioxidative properties were considered, seventeen of the twenty-two compounds present in the four DETa collections showed a positive correlation (see Supplementary Table S2). Among them, eleven phenolics evidenced Pearson correlations (with r values > 0.5) in relation to their antioxidative capability: caffeic, caftaric, ellagic, and rosmarinic acid; epicatechin; gallocatechin gallate; ginnalin A; myricetin; naringin; OH-tyrosol; and quercetin (Table 3).

Within these phenolics, the presence of ginnalin A and naringin is remarkable, since these compounds evidenced the highest correlation values, with r = 0.96 and r = 0.89, respectively (very strong Pearson correlation values). Ginnalin A is a phenolic molecule previously reported in aqueous preparations of *T. absinthioides* [21,23] that has demonstrated antioxidative properties in cells, and it promotes the expression of antioxidative enzymes. In addition, ginnalin A has interesting chemopreventive activities in cancer cells, suppressing cell proliferation and reducing carcinogenesis [38]. Regarding naringin, it is recognized as one of the main polyphenols present in citric fruits and was previously recognized because of its antioxidative capability and its effects on cancer cell proliferation, angiogenesis, and metastasis [39]. The remaining phytochemicals that evidenced Pearson correlations of r > 0.5 with antioxidative properties in DETa are widely accepted as phenolics present in foods, beverages, and condiments that have a recognized reactive oxygen scavenger potency [40,41,42,43,44,45,46].

### 2.5. Principal Component Analysis

The principal component analysis (PCA) was applied to show the relationships between chemical composition, quantified compound values, and bioassays of the samples (cytotoxicity and antioxidative properties).

**Figure 3:**
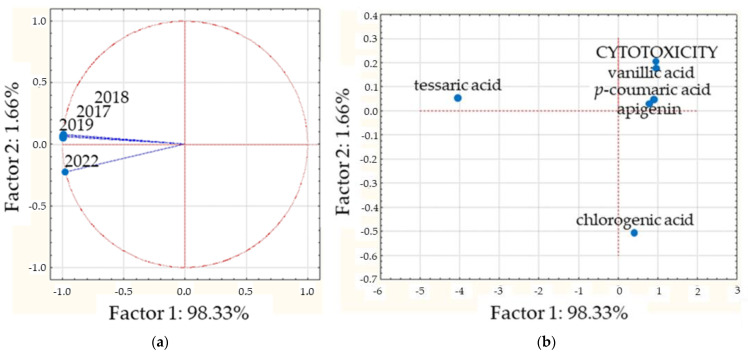
CPA analysis performed on cytotoxicity. (**a**) Projection of the collections on the factor plane. (**b**) Projection of cases on the factor plane.

When cytotoxicity was the property selected, the PCA was realized with four variables, corresponding to the years of collections (2017, 2018, 2019, and 2022) and six cases, including the concentrations of apigenin, chlorogenic acid, *p*-coumaric acid, tessaric acid, and vanillic acid and cytotoxicity values (see Table 2). Two principal components (PCs) were generated: PC1, accounting for 98.33% of the variation; and PC2, accounting for 1.66%; together, they explain 99.99% of the total variation (Supplementary Figure S1a). When the projection on the factor plane of collections is seen (Figure 3a), they present similar, almost equal, projection values on factor 1. The values obtained were −0.99 for the 2017, 2018, and 2019 collections and −0.97 for the 2022 collection, indicating that only slight differences between the collections are present when cytotoxicity is the explored variable. On the other hand, when projections of the major correlated compounds are analyzed on PC1 (Figure 3b), only tessaric acid evidenced a marked difference with regard to the values estimated for the rest of the compounds. While the cytotoxicity value in the PC1 coordinate is 0.98, the values are 0.95 for vanillic acid, 0.87 for *p*-coumaric acid, 0.81 for apigenin, and 0.41 for chlorogenic acid. Because of the variability explained by PC2 is too small (only 1.66%), it could be considered that these compounds are strongly similar in terms of their cytotoxicity. Owing to the results obtained by the correlation analysis applied, these compounds could be proposed as bioactive cytotoxicity markers for all DETa collections. Additionally, tessaric acid could be considered a chemical species marker that is essential for ensuring the authenticity of *T. absinthioides* preparations.

**Figure 4:**
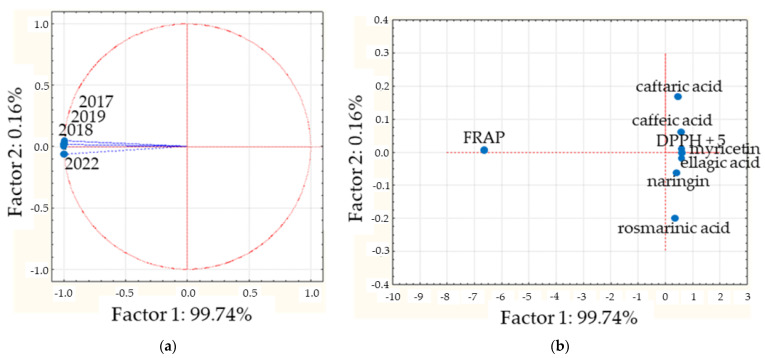
CPA analysis performed on antioxidative properties. (**a**) Projection of the collections on the factor plane. (**b**) Projection of cases on the factor plane. The blue point that indicates “DPPH + 5” refers to DPPH, epicatechin, OH-tyrosol, gallocatechin gallate, quercetin, and ginnalin A.

To analyze the antioxidative activity relative to the chemical composition, the PCA considered four variables, corresponding to the years of collections (2017, 2018, 2019, and 2022) and thirteen cases, including the compounds caffeic, caftaric, ellagic, and rosmarinic acids; epicatechin; gallocatechin gallate; ginnalin A; myricetin; naringin; OH-tyrosol; and quercetin; quantified by DPPH and FRAP values (see Table 3). The two principal components explain almost 100% of the variation; PC1 explains 99.74%, while PC2 explains 0.16% (Supplementary Figure S1b). The projection on the factor plane of the harvests presents almost identical values for PC1 (Figure 4a), and the values obtained were 0.998 for the 2017 collection, 0.999 for 2018, 0.999 for 2019, and 0.998 for 2022; indicating high similarities between the DETa samples vs. antioxidative activity. In Figure 4b, it is possible to observe that only the projection of FRAP is notoriously different in PC1. Regarding the compounds, considering the value of PC2 (0.16%), all of them are similar for DPPH (PC1: 0.627). The individual values obtained were: epicatechin, 0.625; OH-tyrosol, 0.625; gallocatechin gallate, 0.618; quercetin, 0.611; myricetin, 0.597; ellagic acid, 0.587; ginnalin A, 0.583; caffeic acid, 0.574; caftaric acid, 0.436; naringin, 0.434; and rosmarinic acid, 0.322. Consequently, these phenolics could be considered markers for the antioxidative capability of the DETa collections.

In summary, in the current study, through the analysis of DETa botanical preparations, it was possible to identify five compounds related to cytotoxicity markers—apigenin, chlorogenic acid, tessaric acid, *p*-coumaric acid, and vanillic acid—and eleven compounds linked to antioxidative capabilities—naringin, gallocatechin gallate, ginnalin A, myricetin, epicatechin, OH-tyrosol, quercetin, caffeic acid, caftaric acid, ellagic acid, and rosmarinic acid. In addition, tessaric acid should also be considered a species analytical marker.

Based on this study’s results, the sixteen mentioned compounds represent a set of bioactive phytochemicals that are able to interact synergistically to ensure the demonstrated anticancer activities of DETa, being part of the synergistic network of compounds that characterize the activity of botanical preparations.

## 3. Materials and Methods

### 3.1. Chemicals

Standards of apigenin (≥95%), caffeic acid (99%), caftaric acid (≥97%), (+)-catechin (≥99%), chlorogenic acid (≥95%), ellagic acid (≥95%), (−)-epicatechin (≥95%), ferulic acid (≥99), gallic acid (GA, >95%), (−)-gallocatechin gallate (≥99%), ginnalin A (>97%), hesperetin (≥95%), hydroxytyrosol (≥99.5%), kaempferol-3-glucoside (≥99%), luteolin (≥98%), myricetin (≥96%), naringin (≥95%), naringenin (≥95%), *p*-coumaric acid (99%), phloridzin dehydrate (99%), procyanidin B1 (≥90%), procyanidin B2 (≥92%), quercetin (95%), quercetin 3-β-D-glucoside (≥90%), rosmarinic acid (98%), rutin trihydrate (99%), sinaptic acid (≥95%), syringic acid (≥95%), *trans*-cinnamic acid (≥95%), *trans*-piceatannol (>95%), and vanillic acid (≥97%) were purchased from Sigma-Aldrich (St. Louis, MO, USA). The OH-tyrosol (≥99.5%) standard was obtained from Fluka (Buchs, Switzerland). Tessaric acid (≥99%) was previously isolated from the aerial parts of *T. absinthioides*, and the proton ^1^H-NMR spectrum was described by Donadel et al. [47] (Supplementary Figure S2). HPLC-grade acetonitrile (MeCN), ethanol (EtOH), methanol (MeOH), and formic acid (FA) were acquired from Mallinckrodt Baker (Inc., Pillispsburg, NJ, USA). Ultrapure water (H_2_O) was obtained from a Milli-Q system (Millipore, Billerica, MA, USA). Commercial Folin–Ciocalteu (FC) reagent and 2,2-Diphenyl-1-picrylhydrazyl (DPPH) were purchased from Sigma-Aldrich.

### 3.2. Plant Material and Decoction Preparation

*T. absinthioides* (Hook. & Arn.) DC. plants were collected during December in the years 2017, 2018, 2019, and 2022 from Mendoza, Argentina (32°89′79.310″ S, 68°87′57.630″ W). The specimens were deposited in the Mendoza Ruiz Leal herbarium under voucher identification number MERL 65309. To obtain the *T. absinthioides* decoctions (DETa), the leaves of each collection (2017, 2018, 2019, and 2022) were washed with running water and 1% of sodium hypochlorite (*v*/*v*), rinsed with distilled water, and dried at 22–24 °C in a ventilated room, protected from direct solar irradiation for 2 weeks until the leaves reached a constant weight. Then, the dry leaves were finely ground and stored at −20 °C until use. To prepare the decoctions, 50 g of this vegetal material was placed in 1 L of distilled water (5% *p*/*v)*, boiled for 10 min, and filtered. Then, each decoction was sterilized by passing through a 0.22 μm pore size filter. The dry weight of each decoction was calculated independently in triplicate three times by placing 1 mL of DETa into a sterile Eppendorf at 37 °C and using a cultivation stove for 2 weeks until the samples reached a constant weight. The obtained yields were as follows: for 2017: 9.71 ± 0.04 mg/mL; for 2018: 9.51 ± 0.04 mg/mL; for 2019: 8.85 ± 0.05 mg/mL; and for 2022: 9.20 ± 0.02 mg/mL. After the determination of soluble solids, the sample concentration was standardized (diluted with sterile MilliQ water) to obtain the same final concentration of 8.8 mg/mL (dry weight/mL of DETa).

### 3.3. Chemical Analyses of the Acquired T. absinthioides Decoction 

#### 3.3.1. Determination of Phytochemical Profile and Quantification

Determination of the chemical profile and the simultaneous quantification of the compounds present in DETa were conducted by ultra-high-performance liquid chromatography coupled with a diode array and fluorescence detectors (UHPLC-DAD and UHPLC-DAD-FL, respectively). In addition, an ultra-high-performance liquid chromatography-coupled triple quadrupole mass spectrometer (UPLC-MS/MS) was employed for the tessaric acid quantification and structural confirmation based on ^1^H-NMR spectra.

For the UHPLC-DAD analysis, the methodology previously reported by Soto et al. [48] with a few modifications was used. A Shimadzu LC SIL30 (Chiyoda-ku, Tokyo, Japan) system with a C18 column was employed for the following phenolic compounds: apigenin, catechin, chlorogenic acid, luteolin, p-coumaric acid, rutin, synaptic acid, *trans*-cinnamic acid, OH-tyrosol, and vanillic acid (separation conditions are presented in Supplementary Table S3, and representative chromatograms are presented in Supplementary Figure S3). On the other hand, for the UHPLC-DAD-FL analysis, a Dionex Ultimate 3000 system (Sunnyvale, CA, USA) with a C18 column was used, following the methodology described by Ferreyra et al. [49] (separation conditions are presented in Supplementary Table S3, and representative chromatograms are presented in Supplementary Figure S4). This was utilized for the determination of caffeic acid, caftaric acid, ellagic acid, epicatechin, ferulic acid, gallocatechin gallate, ginnalin A, hesperetin, kaempferol-3-glucoside, myricetin, naringin, naringenin, OH-tyrosol, phloridzin, procyanidin B1 and B2, quercetin, quercetin-3-glucoside, rosmarinic acid, syringic acid, and *trans*-piceatannol. For both methodologies, the compounds were identified by comparison of their retention times with those of authentic standards. Standard curves were constructed using commercial reference compounds. Data were expressed as µg/mL of DETa. All analyses were conducted in triplicate.

The UPLC-MS/MS analysis was performed on an ACQUITY H–Class UPLC (Waters Corp., Milford, MA, USA) equipped with a XEVO TQ-S micro triple quadrupole mass spectrometer with an electrospray ionization (ESI) source and C18 column as described by Ortiz et al. [50] (separation conditions are presented in Supplementary Table S3, and representative chromatograms are presented in Supplementary Figure S5). The calibration curve was constructed by using five standard solutions of tessaric acid (1.1, 2.2, 4.4, 6.6, and 8.8 ppm) in triplicate. The DETa samples were prepared at 44 ppm. The samples were dissolved in a mixture of MeOH-H_2_O (50:50) and filtered through a nylon membrane filter (0.22 µm). The data were acquired in the ESI+ mode using the multiple-reaction monitoring (MRM) function of two channels (transitions 249.00 > 203.00; 249.00 > 231.00). The capillary, cone, and collision energies were 3.00 kV, 33.94 V, and 20 eV, respectively. MassLynx Software V4.2 (TargetLynx™, Waters, Milford, MA, USA) was used for the data processing and calibration curve acquisition.

#### 3.3.2. Determination of Total Phenolic Content

The TPC was determined by the colorimetric method using Folin–Ciocalteu as described by Heldrich [51] and modified by Luna et al. (2018). The DETa samples (10 μL) were diluted 4-fold and mixed with MilliQ water (150 μL), Folin–Ciocalteu reagent (12.5 μL), and 20% (*w*/*v*) sodium carbonate solution (37.5 μL). Then, the mixtures were incubated at room temperature for 20 min. The absorbance was measured at a wavelength of 765 nm using a microplate reader (ThermoScientific Multiscan, Waltham, MA, USA). The TPC was determined by linear regression from a calibration plot constructed using gallic acid (0–425 µg/mL) and expressed as µg of gallic acid equivalents (GAE) per mL of DETa (µg GAE/mL DETa).

### 3.4. Bioassays

#### 3.4.1. Cytotoxicity Assay

The MCF-7 human breast adenocarcinoma cell line (ATCC, Manassas, VA, USA) was selected for determining the cytotoxicity effects of DETa samples. Cells were cultured as a monolayer in DMEM (Gibco, Miami, FL, USA) containing 10% *v*/*v* fetal bovine serum (Internegocios, Córdoba, Argentina), 100 IU/mL penicillin, 100 μg/mL streptomycin (Gibco, USA), and 3.7 mg/mL NaHCO_3_ (Sigma-Aldrich, USA). Cells were grown in a humidified atmosphere containing 5% CO_2_ at 37 °C.

Cytotoxicity was determined by the MTT method ((3-(4,5-dimethylthiazol-2-yl)-2,5-diphenyltetrazolium bromide)), which is a colorimetric assay for assessing cell metabolic activity. Briefly, in 96-well microplates, 3.5 × 10^3^/100 μL MCF-7 cells were seeded; after 24 h, the medium was changed to fresh medium containing DETa treatments at the indicated doses and doxorubicin (Onkostatil^®^, Microsules, Buenos Aires, Argentina) as a positive and inter-assay control. After 72 h of treatment, the medium was replaced by the MTT solution, the plates were incubated for an additional 4 h, the MTT solution was removed, and DMSO was added to dissolve the formazan crystals. The optical density was measured at a wavelength of 570 nm using a microplate reader (Thermo Scientific Multiscan, Waltham, MA, USA). Untreated cells (without DETa) were used to represent 100% viability (control), and the other values were calculated accordingly. The assays were performed three times in triplicate for each DETa sample. The obtained median dose for doxorubicin (Dm) as an inter-assay and positive control was 210.40 ± 6.73 ng/mL.

#### 3.4.2. Antioxidative Activity

The antioxidative activity of DETa samples was evaluated by DPPH and FRAP assays.

DPPH scavenging activity: The free radical scavenging effect of the samples was evaluated by the DPPH assay, according to the procedure described by Brand-Williams et al. [52] and modified by Luna et al. [53]. Scavenging activities were evaluated at 517 nm in a Multiskan FC microplate photometer (Thermo Scientific, USA). Quercetin was used as the reference compound. The DETa concentration providing 50% of radical scavenging activity (EC_50_) was calculated by plotting the inhibition percentage and expressed in µg/mL. Analyses were performed in triplicate, and values were reported as the mean ± SD.

Ferric-reducing antioxidant power (FRAP) assay: The assay was performed in accordance with Benzie and Strain [54], with some modifications by Luna et al. [53]. Briefly, the FRAP solution was freshly prepared by mixing 10 mL of acetate buffer 300 mM at pH 3.6, 1 mL of ferric chloride hexahydrate 20 mM dissolved in distilled water, and 1 mL of 2,4,6-tris(2-pyridyl)-s-triazine 10 mM dissolved in HCl 40 mM. Ten µL of DETa sample solution was mixed with 190 µL of the FRAP solution in triplicate in 96-well microplates, and the change in absorbance was evaluated at 620 nm using a Multiskan FC microplate photometer (Thermo Scientific, USA). Results were obtained by linear regression from a calibration plot obtained with Trolox equivalents per mL. All samples were analyzed in triplicate, and the data are reported as the mean ± SD. Results are expressed as µg TE/mL of DETa.

### 3.5. Statistical Analysis

All the data were expressed as the mean ± standard error (SEM) of triplicates. When comparison between groups was necessary, they were evaluated using analysis of variance (ANOVA), and the means were compared by the Tukey’s multiple-comparison test, considering significance when *p* ≤ 0.05. Cytotoxic potency was expressed as Dm, which is the median-effect dose (ED_50_) that reduces proliferation by 50%. The multivariate analyses were performed using the Pearson correlation coefficient and principal component analysis (PCA). The GraphPad Prism 6.0 (Graph Pad Software Inc., San Diego, CA, USA), CompuSyn 1.0 (ComboSyn, Inc., Houston, TX, USA), XLSTAT Excel 2016 software (Addinsoft, New York, NY, USA), and Statistica 7 (StatSoft Inc., Tulsa, OK, USA) software were used to perform the analyses.

#### 3.5.1. Pearson Correlation

The correlation matrix was calculated, giving the correlation coefficients between each pair of variables tested, by the use of the XLSTAT software (Addinsoft, USA, 2011). Pearson’s correlation coefficients (r) between compound contents in the DETa and the cytotoxic and antioxidative activities were determined. Only compounds quantified in the four DETa samples were considered. To describe the strength of the correlation, the Evans’ correlation guide was followed; values of r = 0.01 to 0.19 are considered very weak, values from 0.20 to 0.39 are weak, values from 0.40 to 0.59 are moderate, values from 0.60 to 0.79 are strong, and values from 0.80 to 1.0 represent a very strong correlation [55].

#### 3.5.2. Principal Component Analysis (PCA)

PCA was implemented in the Statistica software to classify the different collections based on their bioactive compounds and biological properties. Only compounds which positively correlated with cytotoxicity, DPPH, or FRAP (pairwise Pearson; correlation coefficient values r ≤ 0.5) were considered, and the calculated phytochemical concentration of each DETa sample was analyzed. The data set regarding cytotoxicity consists of a matrix of the order 4 × 6, where the columns represent the harvest (2017, 2018, 2019 and 2022), and the rows comprise the data for the five phytochemicals concentrations that correlate with the cytotoxicity estimated by MTT. Meanwhile, the data set related to the antioxidative activity consists of a matrix of the order 4 × 13, where the columns represent the DETa collection samples, and the columns include the concentrations of compounds that correlate with the antioxidative capacity estimated by the DPPH and FRAP.

## 4. Conclusions

In this study, a correlation analysis was conducted to examine the relationship between the compounds quantified and the cytotoxic activity and antioxidative properties of DETa sample collections obtained during four different years to determine their potential oncological applications. Cytotoxic as well as antioxidative activities did not vary among the collections. Regarding the quantified compounds, the flavone apigenin, naringin, gallocatechin gallate, ginnalin A, myricetin, epicatechin, OH-tyrosol, quercetin, chlorogenic acid, tessaric acid, *p*-coumaric acid, vanillic acid, caffeic acid, caftaric acid, ellagic acid, and rosmarinic acid correlated as bioactive and chemical markers. Moreover, tessaric acid could be established as a species marker.

In accordance with our knowledge, the current work is the first report that correlates the chemical composition of DETa with its cytotoxic and antioxidative properties. The presented results support the anti-oncological properties previously described for aqueous preparations of *T. absinthioides* and encourage additional exploration of its potential application as a promising anticancer agent. In addition, through the description of bioactive and chemical markers, the present study provides novel starting points for expanding the knowledge of this botanical plant derivative and its application for cancer control and treatment.

## Figures and Tables

**Table 1:** Concentrations of compounds quantified by UHPLC-DAD, UHPLC-DAD-FLD, and UPLC-MS/MS from the DETa collections (µg/mL).

Compounds (*n* = 22)	2017		2018		2019		2022	
	Mean	SD	Mean	SD	Mean	SD	Mean	SD
Apigenin ^D^	3.03	0.27	5.9	0.41	3.71	0.18	2.63	0.49
Caffeic acid ^F^	22.65	1.93	1.01	0.05	0.46	0.03	1.74	0.06
Caftaric acid ^F^	75.74	2.90	5.80	0.14	3.82	0.17	7.69	0.30
Catechin ^D^	4.91	0.40	1.47	0.26	9.90	0.53	2.41	0.06
Chlorogenic acid ^D^	11.10	1.12	13.10	0.73	8.37	0.61	10.27	0.96
Ellagic acid ^F^	3.50	0.31	4.03	0.46	0.18	0.01	11.89	0.48
Epicatechin ^F^	0.16	0.01	0.22	0.01	0.14	0.01	0.35	0.01
Ferulic acid ^F^	0.44	0.02	0.21	0.01	0.24	0.01	0.11	0.02
Gall-gallate ^F^	0.88	0.04	0.96	0.03	0.58	0.01	1.90	0.03
Ginnalin A ^F^	8.87	0.76	4.11	0.43	1.51	0.28	7.09	0.81
Hesperetin ^F^	6.53	0.08	3.57	0.14	2.30	0.10	1.96	0.02
Luteolin ^D^	7.29	1.2	8.38	0.41	11.95	0.53	4.56	0.06
Myricetin ^F^	3.17	0.13	2.35	0.09	0.92	0.13	8.33	0.07
Naringin ^F^	26.34	0.68	21.39	0.55	1.92	0.15	44.43	0.96
OH-tyrosol ^F^	0.16	0.01	0.15	0.01	0.10	0.01	0.63	0.04
*p*-Coumaric acid ^D^	0.73	0.02	3.28	0.16	0.03	0.01	2.20	0.06
Quercetin ^F^	2.06	0.15	1.51	0.43	1.84	0.09	2.35	0.35
Quercetin-3-gluc ^F^	5.83	0.40	36.68	2.64	1.47	0.17	45.32	1.41
Rosmarinic acid ^F^	31.22	2.25	20.91	0.40	5.14	0.10	93.22	1.51
Tessaric acid ^M^	326.71	5.19	559.53	6.13	190.01	2.74	29.80	1.85
*trans*-Piceatannol ^F^	0.17	0.01	2.65	0.05	1.27	0.03	1.50	0.02
Vanillic acid ^D^	0.37	0.04	0.58	0.01	0.48	0.03	0.35	0.02

Abreviations: Gall-gallate: gallocatechin gallate; Quercetin-3-gluc: quercetin-3-glucoside. The superscript letters, D, F and M indicate compounds quantified by UHPLC-DAD UHPLC-DAD-FLD, and UPLC MS/MS, respectively.

**Table 2:** Pearson’s positive correlations (r > 0.5) between compounds and their cytotoxicity.

Compound	Apigenin	CQA	*p*-coum ac.	Tessaric ac.	Vanillic ac.	Cytotoxicity
apigenin	1	0.63	0.57	0.87	0.96	0.74
CQA	0.63	1	0.83	0.76	0.41	0.98
*p*-coumaric ac.	0.57	0.83	1	0.43	0.39	0.73
tessaric ac.	0.87	0.76	0.43	1	0.77	0.89
vanillic ac.	0.96	0.41	0.39	0.77	1	0.54
cytotoxicity	0.74	0.98	0.73	0.89	0.54	1

Abbreviations: CQA; chlorogenic acid; ac: acid.

**Table 3:** Pearson’s positive correlations (r > 0.5) between compounds and their antioxidative capability measured by the DPPH and FRAP assays.

Compound	Caff	Caft	Ella	Epi	Gall	Gin	Myr	Nar	O-t	Que	Ros	DPPH	FRAP
Caffeic ac.	1	1	−0.14	−0.34	−0.19	0.75	−0.06	0.16	−0.23	0.26	−0.06	0.56	0.17
Caftaric ac.	1	1	−0.14	−0.34	−0.64	−0.52	−0.57	−0.77	−0.48	−0.01	−0.56	0.56	0.16
Ellagic ac.	−0.14	−0.14	1	0.98	1	0.55	0.99	0.95	0.97	0.67	0.98	0.74	0.36
Epicatechin	−0.34	−0.34	0.98	1	0.98	0.37	0.94	0.87	0.96	0.90	0.94	0.58	0.28
Galloc-gall	−0.19	−0.64	1	0.98	1	0.50	0.99	0.92	0.98	0.68	0.99	0.71	0.39
Ginnalin A	0.75	−0.52	0.55	0.37	0.50	1	0.59	0.77	0.43	0.59	0.58	0.96	0.29
Myricetin	−0.06	−0.57	0.99	0.94	0.99	0.59	1	0.93	0.98	0.78	1	0.78	0.51
Naringin	0.16	−0.77	0.95	0.87	0.92	0.77	0.93	1	0.86	0.64	0.93	0.89	0.28
OH-tyrosol	−0.23	−0.48	0.97	0.96	0.98	0.43	0.98	0.86	1	0.76	0.98	0.65	0.54
Quercetin	0.26	−0.01	0.67	0.90	0.68	0.59	0.78	0.64	0.76	1	0.79	0.74	0.91
Rosmar. ac	−0.06	−0.56	0.98	0.94	0.99	0.58	1	0.93	0.98	0.79	1	0.78	0.52
FRAP	0.56	0.56	0.74	0.58	0.71	0.96	0.78	0.89	0.65	0.74	0.78	1	0.42
DPPH	0.17	0.16	0.36	0.28	0.39	0.29	0.51	0.28	0.54	0.91	0.52	0.42	1

Abbreviations: Caff: caffeic acid; Caft: caftaric acid; Ella: ellagic acid; Epi: epicatechin; Gall and Gallo-gall: gallocatechin gallate; Gin: ginnalin A; Myr: myricetin; Nar: naringin; O-t: OH-tyrosol; Que: quercetin; Ros and Rosmar ac.: rosmarinic acid.

## Data Availability

After publication, data will be placed at the “Repositorio de datos del CONICET” (https://ri.conicet.gov.ar/).

## Supplementary Materials

The following supporting information can be downloaded at: https://www.mdpi.com/article/10.3390/plants13213062/s1, Table S1: Occasional DETa phytochemical concentrations; Table S2: Complete PEARSON correlations of phytochemicals and biological properties; Table S3: Chromatographic separation conditions; Figure S1: CPA eigenvalues; Figure S2: ^1^H-NMR spectra—Tessaric acid; Figure S3: UHPLC-DAD demonstrative chromatograms of standards and samples; Figure S4: UHPLC-DAD-FLD representative chromatograms. In (a) and (c) phenolic compounds in standard solution. In (b) and (d) phenolic compounds in DETa; Figure S5: UPLC-MS/MS Tessaric acid chromatograms. In (a) Tessaric acid in standard solution. In (b) Tessaricc acid in DETa (b).
