# Supplementary material for: Correlation Analysis Among the Chemical Composition and Cytotoxic and Antioxidative Activities of a Tessaria absinthioides Decoction for Endorsing Its Potential Application in Oncology
Source: Plants (Basel). 2024 Oct 31;13(21):3062. doi: 10.3390/plants13213062 (PMC11548503; doi:10.3390/plants13213062)
Supplement: Supplementary file 1 [file plants-13-03062-s001.zip › Supplementary Figure FS2_1H-NMR spectra & structure Tessaric acid..pdf]

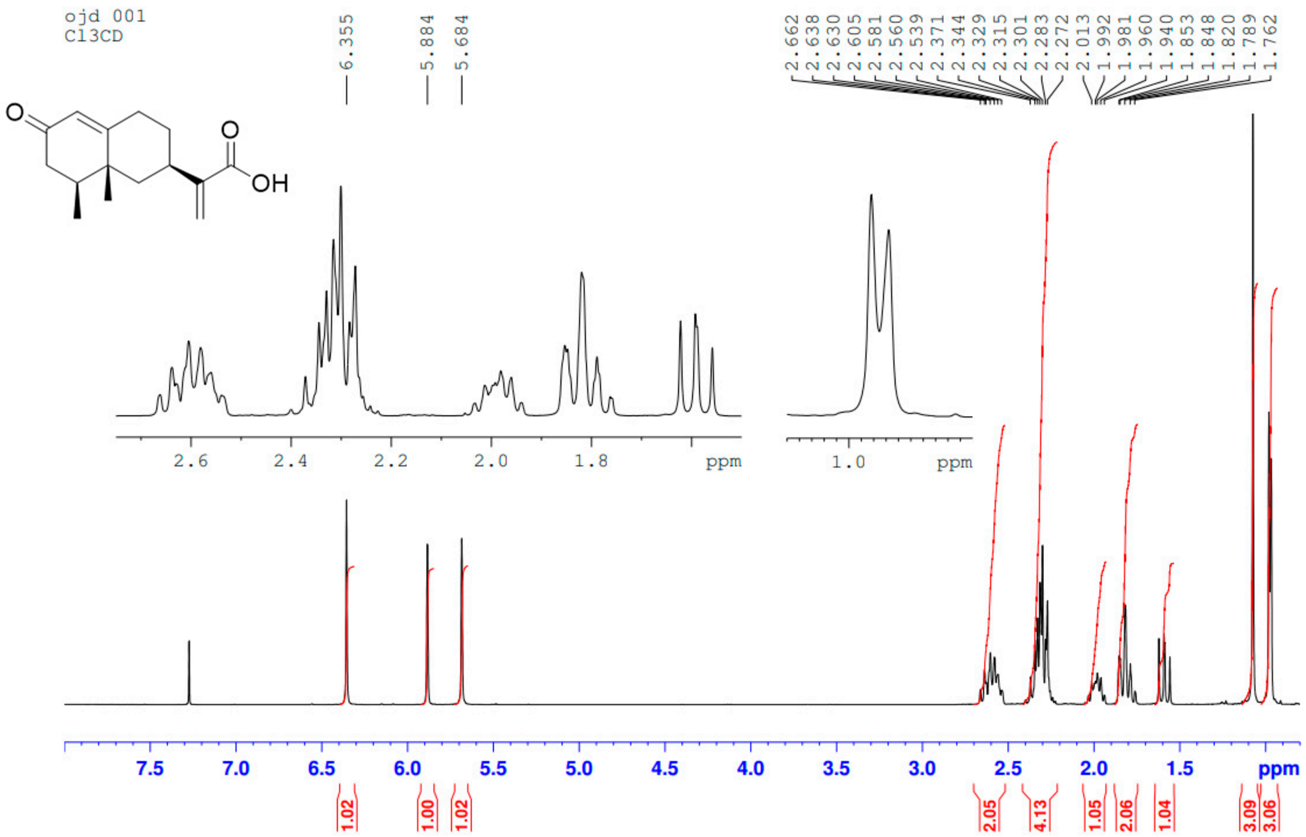

Suplementary Figure S2. ¹H-NMR spectrum – Tessaric acid. Tessaric acid, 1 H NMR (200 MHz, CDCl3) d 6.33 (br s, 1H, H13), d 5.66 (br s, 1H, H130), d 5.88 (br s, 1H, H1), d 0.98 (d, J = 7 Hz, 3H, H15), d 1.10 (s, 3H, H14), d 2.58 (m, 1H, H7); Donadel *et al.*, 2005.
