# Supplementary material for: Correlation Analysis Among the Chemical Composition and Cytotoxic and Antioxidative Activities of a Tessaria absinthioides Decoction for Endorsing Its Potential Application in Oncology
Source: Plants (Basel). 2024 Oct 31;13(21):3062. doi: 10.3390/plants13213062 (PMC11548503; doi:10.3390/plants13213062)
Supplement: Supplementary file 1 [file plants-13-03062-s001.zip › Supplementary Figure FS3_UHPL-DAD Chromatograms.pdf]

plants

MDPI

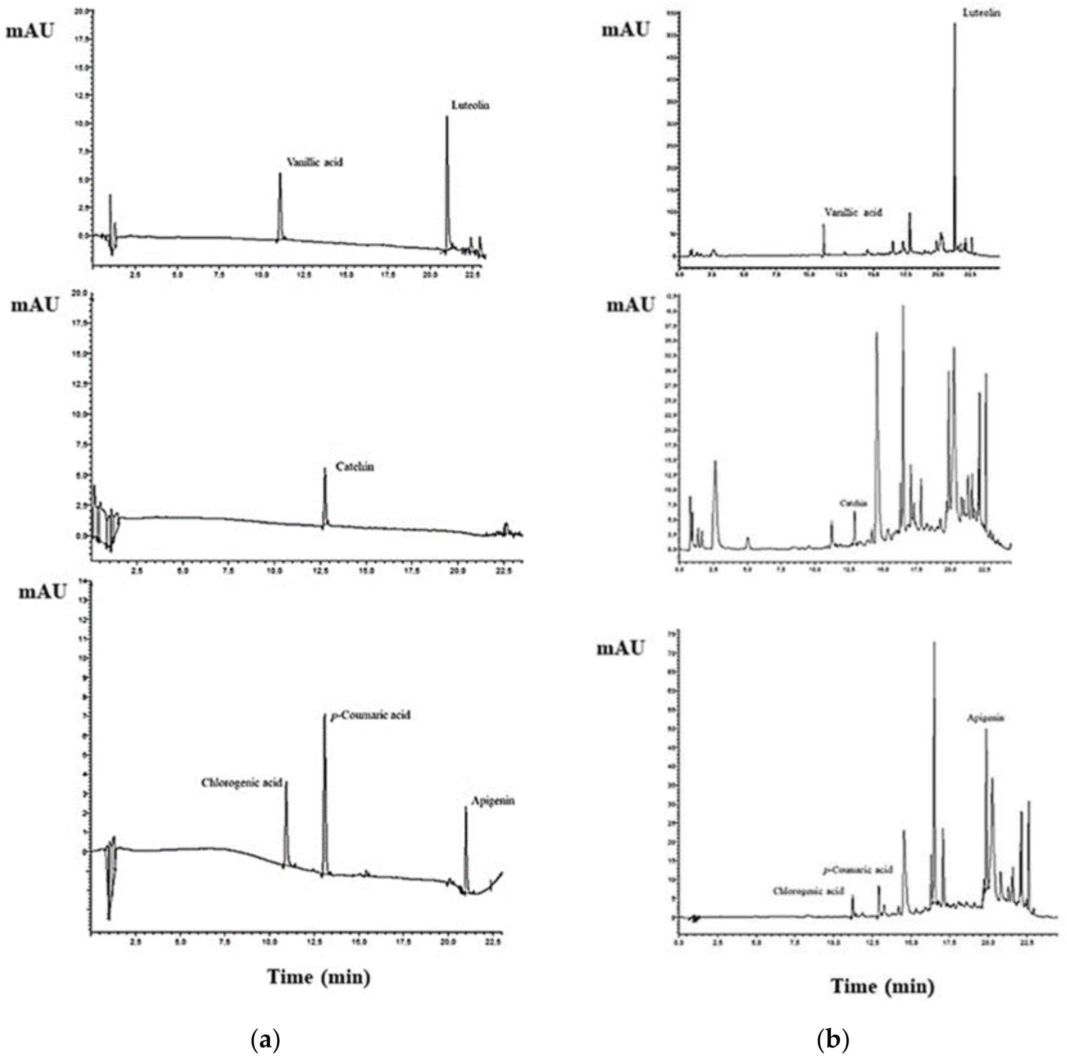

Suplementary Figure S3. Figure . UHPLC-DAD representative chromatograms. In (a) phenolic compounds in standard solution. In (b) phenolic compounds in DETa (b).
