# Supplementary material for: Correlation Analysis Among the Chemical Composition and Cytotoxic and Antioxidative Activities of a Tessaria absinthioides Decoction for Endorsing Its Potential Application in Oncology
Source: Plants (Basel). 2024 Oct 31;13(21):3062. doi: 10.3390/plants13213062 (PMC11548503; doi:10.3390/plants13213062)
Supplement: Supplementary file 1 [file plants-13-03062-s001.zip › Supplementary Figure FS4_UHPL-DAD-FLD Chromatograms.pdf]

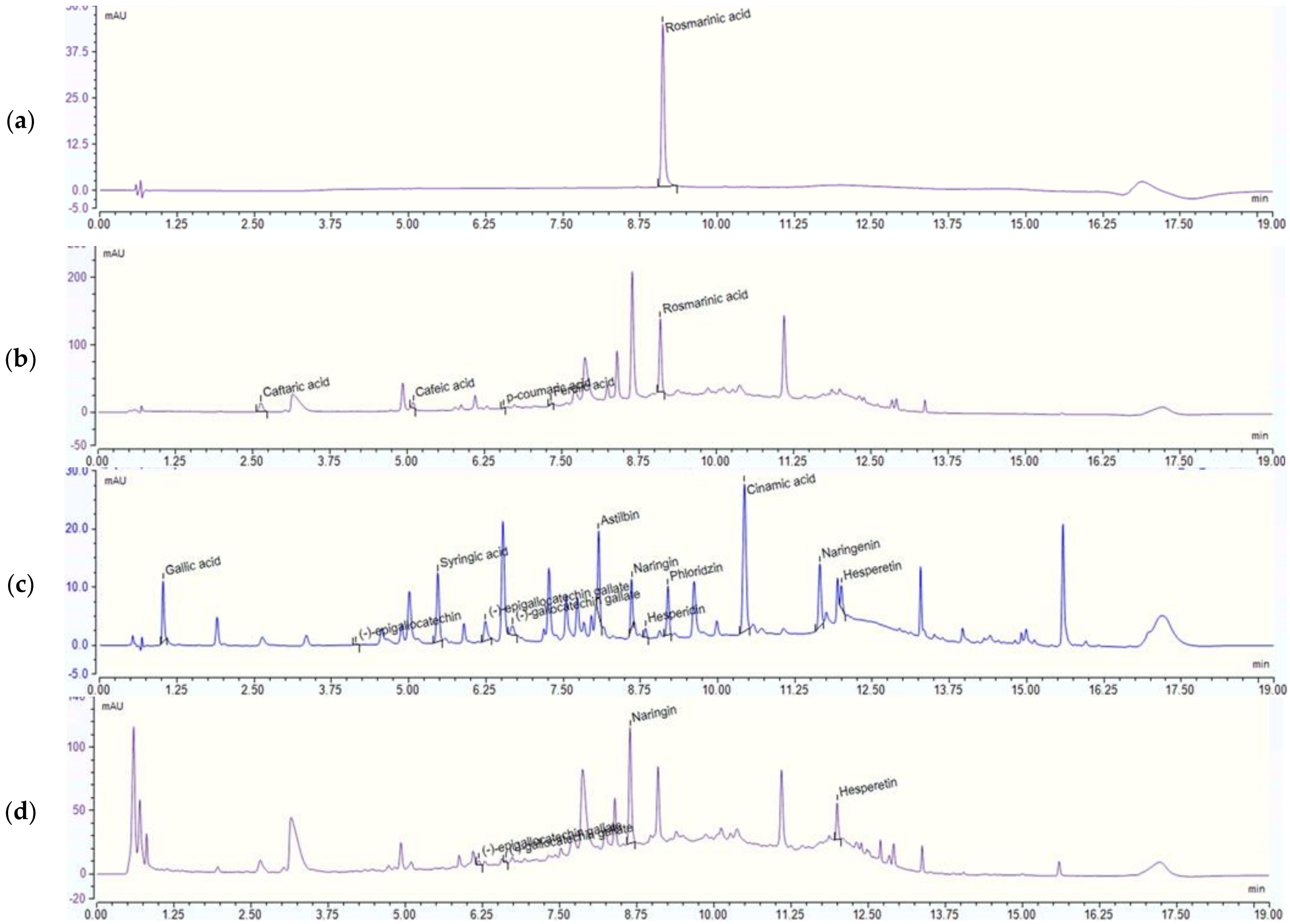

Suplementary Figure S4: UHPLC-DAD-FLD representative chromatograms. In (a) and (c) phenolic compounds in standard solution. In (b) and (d) phenolic compounds in DETa.
