# Supplementary material for: Correlation Analysis Among the Chemical Composition and Cytotoxic and Antioxidative Activities of a Tessaria absinthioides Decoction for Endorsing Its Potential Application in Oncology
Source: Plants (Basel). 2024 Oct 31;13(21):3062. doi: 10.3390/plants13213062 (PMC11548503; doi:10.3390/plants13213062)
Supplement: Supplementary file 1 [file plants-13-03062-s001.zip › Supplementary Figure FS5_UPLC-MS Chromatograms.pdf]

plants

MDPI

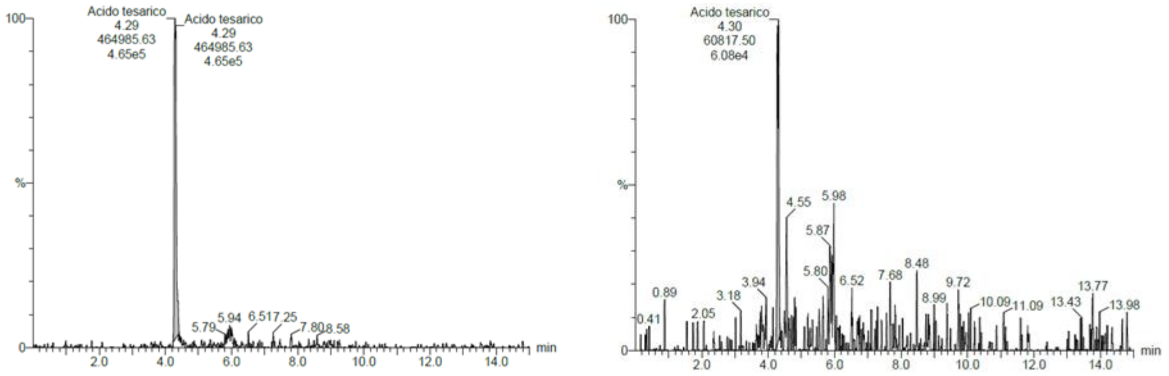

(**a**) (**b**)

Suplementary Figure S5: UPLC-MS/MS Tessaric acid chromatograms. In (a) Tessaric acid in standard solution. In (b) Tessaricc acid in DETa (b).
