# Supplementary material for: Correlation Analysis Among the Chemical Composition and Cytotoxic and Antioxidative Activities of a Tessaria absinthioides Decoction for Endorsing Its Potential Application in Oncology
Source: Plants (Basel). 2024 Oct 31;13(21):3062. doi: 10.3390/plants13213062 (PMC11548503; doi:10.3390/plants13213062)
Supplement: Supplementary file 1 [file plants-13-03062-s001.zip › Supplementary Table TS1_Sporadical Phytochem.pdf]

Supplementary Table S1. Occasional DETa phytochemicals concentrations.

Phenolic group	Compound (n=10)	Harvest year	Concentration (μg/mL)
Phenolic acids	synaptic	2017	0.41
	syringic	2019	0.02
	trans-cinnamic	2017	0.03
Phenylethanoid	tyrosol	2019	0.19
Flavonoids	kaempferol-3-glucoside	2019	0.02
	naringenin	2017 and 2018	0.05; 0.30
	phloridzin	2019	0.01
	procyanidin B1	2019	0.03
	procyanidin B2	2019	0.03
	rutin	2017	0.01
