# Supplementary material for: Correlation Analysis Among the Chemical Composition and Cytotoxic and Antioxidative Activities of a Tessaria absinthioides Decoction for Endorsing Its Potential Application in Oncology
Source: Plants (Basel). 2024 Oct 31;13(21):3062. doi: 10.3390/plants13213062 (PMC11548503; doi:10.3390/plants13213062)
Supplement: Supplementary file 1 [file plants-13-03062-s001.zip › Supplementary Table TS2-PEARSON Complete.pdf]

plants MDPI

Supplementary Table S2. Complete PEARSON correlations of phytochemicals and biological properties.

Comp	Api	Caf	Cta	Cat	CQA	Ela	Epi	Fea	Gag	GiA	Hes	Lut	Myr	Nar	OHt	*p*Co	Que	Q3g	Roa	Tea	*t*Pi	Vaa	CYT	DPPH	FRAP
**Api**	1	-0,38	-0,37	-0,29	0,63	-0,39	-0,26	-0,11	-0,40	-0,49	-0,03	0,37	-0,54	-0,38	-0,52	0,57	-0,95	0,19	-0,55	0,87	0,78	0,96	0,74	-0,58	-0,97
**Caf**	-0,38	1	1,00	0,00	0,15	-0,14	-0,34	0,90	-0,19	0,75	0,94	-0,21	-0,06	0,16	-0,23	-0,35	0,26	-0,46	-0,06	0,13	-0,80	-0,49	0,17	0,56	0,17
**Cfa**	-0,37	1,00	1	0,00	0,15	-0,14	-0,34	0,90	-0,19	0,74	0,94	-0,21	-0,07	0,15	-0,24	-0,35	0,25	-0,46	-0,07	0,14	-0,80	-0,49	0,17	0,56	0,16
**Cat**	-0,29	0,00	0,00	1	-0,85	-0,68	-0,67	0,28	-0,64	-0,52	-0,10	0,78	-0,57	-0,77	-0,48	-0,92	-0,01	-0,85	-0,56	-0,31	-0,45	-0,05	-0,71	-0,56	0,40
**CQA**	0,63	0,15	0,15	-0,85	1	0,18	0,18	0,07	0,14	0,35	0,39	-0,40	0,06	0,37	-0,06	0,83	-0,44	0,52	0,04	0,76	0,46	0,41	0,98	0,25	-0,77
**Ela**	-0,39	-0,14	-0,14	-0,68	0,18	1	0,98	-0,56	1,00	0,55	-0,30	-0,92	0,99	0,95	0,97	0,52	0,67	0,83	0,98	-0,49	0,13	-0,53	-0,04	0,74	0,36
**Epi**	-0,26	-0,34	-0,34	-0,67	0,18	0,98	1	-0,72	0,98	0,37	-0,47	-0,84	0,94	0,87	0,96	0,60	0,56	0,90	0,94	-0,46	0,31	-0,38	-0,03	0,58	0,28
**Fea**	-0,11	0,90	0,90	0,28	0,07	-0,56	-0,72	1	-0,60	0,38	0,93	0,23	-0,50	-0,29	-0,63	-0,50	-0,11	-0,74	-0,50	0,35	-0,71	-0,16	0,18	0,14	-0,05
**Fag**	-0,40	-0,19	-0,19	-0,64	0,14	1,00	0,98	-0,60	1	0,50	-0,36	-0,90	0,99	0,92	0,98	0,50	0,68	0,82	0,99	-0,53	0,14	-0,53	-0,08	0,71	0,39
**GiA**	-0,49	0,75	0,74	-0,52	0,35	0,55	0,37	0,38	0,50	1	0,62	-0,81	0,59	0,77	0,43	0,14	0,59	0,21	0,58	-0,12	-0,53	-0,70	0,22	0,96	0,29
**Hes**	-0,03	0,94	0,94	-0,10	0,39	-0,30	-0,47	0,93	-0,36	0,62	1	-0,08	-0,27	0,02	-0,45	-0,17	-0,08	-0,43	-0,28	0,47	-0,57	-0,17	0,46	0,39	-0,19
**Lut**	0,37	-0,21	-0,21	0,78	-0,40	-0,92	-0,84	0,23	-0,90	-0,81	-0,08	1	-0,91	-1,00	-0,82	-0,52	-0,63	-0,72	-0,91	0,28	0,05	0,58	-0,19	-0,91	-0,26
**Myr**	-0,54	-0,06	-0,07	-0,57	0,06	0,99	0,94	-0,50	0,99	0,59	-0,27	-0,91	1	0,93	0,98	0,38	0,78	0,72	1,00	-0,60	-0,02	-0,66	-0,16	0,78	0,51
**Nar**	-0,38	0,16	0,15	-0,77	0,37	0,95	0,87	-0,29	0,92	0,77	0,02	-1,00	0,93	1	0,86	0,53	0,64	0,74	0,93	-0,32	-0,02	-0,58	0,16	0,89	0,28
**OHt**	-0,52	-0,23	-0,24	-0,48	-0,06	0,97	0,96	-0,63	0,98	0,43	-0,45	-0,82	0,98	0,86	1	0,35	0,76	0,73	0,98	-0,68	0,07	-0,60	-0,27	0,65	0,54
***p*Co**	0,57	-0,35	-0,35	-0,92	0,83	0,52	0,60	-0,50	0,50	0,14	-0,17	-0,52	0,38	0,53	0,35	1	-0,27	0,88	0,37	0,43	0,77	0,39	0,73	0,19	-0,60
**Que**	-0,95	0,26	0,25	-0,01	-0,44	0,67	0,56	-0,11	0,68	0,59	-0,08	-0,63	0,78	0,64	0,76	-0,27	1	0,14	0,79	-0,87	-0,58	-0,97	-0,61	0,74	0,91
**Q3g**	0,19	-0,46	-0,46	-0,85	0,52	0,83	0,90	-0,74	0,82	0,21	-0,43	-0,72	0,72	0,74	0,73	0,88	0,14	1	0,72	-0,04	0,65	0,04	0,35	0,37	-0,17
**Roa**	-0,55	-0,06	-0,07	-0,56	0,04	0,98	0,94	-0,50	0,99	0,58	-0,28	-0,91	1,00	0,93	0,98	0,37	0,79	0,72	1	-0,61	-0,03	-0,66	-0,18	0,78	0,52
**Tea**	0,87	0,13	0,14	-0,31	0,76	-0,49	-0,46	0,35	-0,53	-0,12	0,47	0,28	-0,60	-0,32	-0,68	0,43	-0,87	-0,04	-0,61	1	0,41	0,77	0,89	-0,32	-0,95
***t*Pi**	0,78	-0,80	-0,80	-0,45	0,46	0,13	0,31	-0,71	0,14	-0,53	-0,57	0,05	-0,02	-0,02	0,07	0,77	-0,58	0,65	-0,03	0,41	1	0,76	0,45	-0,44	-0,67
**Vaa**	0,96	-0,49	-0,49	-0,05	0,41	-0,53	-0,38	-0,16	-0,53	-0,70	-0,17	0,58	-0,66	-0,58	-0,60	0,39	-0,97	0,04	-0,66	0,77	0,76	1	0,54	-0,78	-0,88
**CYT**	0,74	0,17	0,17	-0,71	0,98	-0,04	-0,03	0,18	-0,08	0,22	0,46	-0,19	-0,16	0,16	-0,27	0,73	-0,61	0,35	-0,18	0,89	0,45	0,54	1	0,08	-0,87

**DPPH**	-0,58	0,56	0,56	-0,56	0,25	0,74	0,58	0,14	0,71	0,96	0,39	-0,91	0,78	0,89	0,65	0,19	0,74	0,37	0,78	-0,32	-0,44	-0,78	0,08	1	0,42
**FRAP**	-0,97	0,17	0,16	0,40	-0,77	0,36	0,28	-0,05	0,39	0,29	-0,19	-0,26	0,51	0,28	0,54	-0,60	0,91	-0,17	0,52	-0,95	-0,67	-0,88	-0,87	0,42	1

Abreviations: Api: apigenin; Caf: caffeic acid; Cfa: caftaric acid; Cat: catechin; CQA; chlorogenic acid; Ela: ellagic acid; Epi: epicatechin; Fea: ferulic acid; Gag: gallocatechin gallate; GiA; ginnalin A; Hes: hesperetin; Lut: luteolin; Myr: myricetin; Nar: naringin; OHt: OH-tyrosol; *p*Co: *p*-coumaric acid; Que: quercetin; Q3g: quercetin-3-glucoside; Roa: rosmarinic acid; Tea: tessaric acid; *t*Pi: *trans*-piceatannol; Vaa: vanillic acid; CYT: cytotoxicity. Pink highlight indicates correlation between phytochemicals (Pearson, r ≥ 0.95).Blue highlight indicates 0.01-0.39 values between compounds and biological properties, considered as positive very weak or weak correlations. Yellow highlight indicates 0.40-0.59 values, considered as positive moderate correlation. Green highlight indicates 0.60-1 values, considered as positive strong or very strong correlations. Summarized correlation values (Pearson, r > 0.5) are presented in the main text (Tables 2 and 3).
