# Supplementary material for: Correlation Analysis Among the Chemical Composition and Cytotoxic and Antioxidative Activities of a Tessaria absinthioides Decoction for Endorsing Its Potential Application in Oncology
Source: Plants (Basel). 2024 Oct 31;13(21):3062. doi: 10.3390/plants13213062 (PMC11548503; doi:10.3390/plants13213062)
Supplement: Supplementary file 1 [file plants-13-03062-s001.zip › Supplementary Table TS3_UHPLC Conditions.pdf]

Supplementary Table S3. Chromatographic separation conditions.

Method	Conditions	
UHPLC-DAD	Analytical column	UFLC Aqueous C18, 2.1 x 100 mm, 3 µm particle size.
	Mobile phase	A: water acidified with formic acid (0.1% v/v), B: acetonitrile.
	Mobile phase elution	Gradient.
	Gradient program	0 min: 5% B; 4 min: 5% B; 17 min: 30% B; 18 min: 35% B; 19 min: 50% B; 20 min: 95% B, 21 min: 95% B; 24 min: 50% B; 27 min: 5% B; 37 min: 5% B.
	Flow rate	0.4 mL min^{-1}
	Detection	λ=254, 280 and 320 nm.
	Sample volume injection	0.1 µL
	Column temperature	30 °C
UHPLC-DAD-FLD	Analytical column	Kinetex C18, 3.0 x 100 mm, 2.6 µm particle size.
	Mobile phase	A: water acidified with formic acid (0.1% v/v), B: methanol.
	Mobile phase elution	Gradient.
	Gradient program	0-1.7 min: 5% B; 1.7-10 min: 30% B; 10-13.5 min: 95% B; 13.5-15 min: 95% B; 15-16 min: 5% B; 16-19 min: 5% B.
	Flow rate	0.8 mL min^{-1}
	Detection	DAD: λ= 254, 280, 320 and 370 nm<br>FL: excitation wavelength (Ex)= 290 nm; emission (Em) response: 315 nm, 360 and 400 nm.
	Sample volume injection	1 µL
	Column temperature	35 °C
UPLC-MS/MS	Analytical column	BEH C18, 2.1 mm × 50 mm, 1.7 µm particle size.
	Mobile phase	A: water acidified with formic acid (1% v/v), B: methanol.
	Mobile phase elution	Gradient.
	Gradient program	0-2 min: 5% B; 6min: 15% B; 10-13 min: 100% B; 13.10-15 min: 5% B.
	Flow rate	0.8 mL min^{-1}
	Detection	DAD: λ= 254, 280, 320 and 370 nm<br>FL: excitation wavelength (Ex)= 290 nm; emission (Em) response: 315 nm, 360 and 400 nm.
	Sample volume injection	10 µL
	Column temperature	35 °C
